# How patients with neuromyelitis optica spectrum disease accumulate disability

**DOI:** 10.1177/13524585261489039

**Published:** 2026-10-08

**Authors:** Sifat Sharmin, Izanne Roos, Pakeeran Siriratnam, Serkan Ozakbas, Dana Horáková, Eva Kubala Havrdova, Petra Nytrová, Recai Turkoglu, Francesco Patti, Bassem Yamout, Samia J. Khoury, Abdullah Al-Asmi, Guy Laureys, Raed Alroughani, Jeannette Lechner-Scott, Cavit Boz, Ayse Altintas, Aysun Soysal, Sara Eichau, Anneke van der Walt, Vilija Jokubaitis, Helmut Butzkueven, Mastura Monif, Tomas Kalincik

**Affiliations:** 1 CORe, Department of Medicine, University of Melbourne, VIC, Australia; 2 Neuroimmunology Centre, Department of Neurology, Royal Melbourne Hospital, VIC, Australia; 3 Department of Neuroscience, School of Translational Medicine, Monash University, Melbourne, VIC, Australia; 4 Department of Neurology, Alfred Health, Melbourne, VIC, Australia; 5 Department of Neurology, Walton Centre NHS Foundation Trust, Liverpool, UK; 6 Faculty of Medicine, Izmir University of Economics, Turkey; 7 Multiple Sclerosis Research Association, Izmir, Turkey; 8 Department of Neurology and Center of Clinical Neuroscience, First Faculty of Medicine, Charles University, Prague, Czech Republic; 9 Department of Neurology, General University Hospital, Prague, Czech Republic; 10 Department of Neurology, Haydarpasa Numune Training and Research Hospital, Istanbul, Turkey; 11 Department of Medical, Surgical Sciences and Advanced Technologies ‘GF Ingrassia’, University of Catania, Italy; 12 Multiple Sclerosis Centre, AOU Policlinico ‘G Rodolico-San Marco’, University of Catania, Italy; 13 Neurology Institute, Harley Street Medical Center, Abu Dhabi, United Arab Emirates; 14 Nehme and Therese Tohme Multiple Sclerosis Center, American University of Beirut Medical Center, Lebanon; 15 College of Medicine & Health Sciences, Sultan Qaboos University, Muscat, Oman; 16 Department of Neurology, Sultan Qaboos University Hospital, Muscat, Oman; 17 Department of Neurology, University Hospital Ghent, Belgium; 18 Division of Neurology, Department of Medicine, Amiri Hospital, Sharq, Kuwait; 19 Hunter Medical Research Institute, The University of Newcastle, Callaghan, NSW, Australia; 20 Hunter New England Health, John Hunter Hospital, Newcastle, NSW, Australia; 21 Department of Neurology, KTU Medical Faculty Farabi Hospital, Trabzon, Turkey; 22 Department of Neurology, School of Medicine and Koc University Research Center for Translational Medicine (KUTTAM), Koc University, Istanbul, Turkey; 23 Department of Neurology, Bakirkoy Education and Research Hospital for Psychiatric and Neurological Diseases, Istanbul, Turkey; 24 Department of Neurology, Hospital Universitario Virgen Macarena, Sevilla, Spain

**Keywords:** Devic’s disease, cohort study, observational data, longitudinal analysis, relapse history

## Abstract

**Background:**

It is believed that patients with neuromyelitis optica spectrum disease acquire disability exclusively through relapses. However, the possibility of disability accumulation in the absence of relapses has also been suggested. We quantified irreversible disability accumulation in the presence and absence of relapses in neuromyelitis optica spectrum disease.

**Methods:**

We identified patients with neuromyelitis optica spectrum disease in the MSBase registry. Disability-worsening events were required to be sustained until the end of follow-up. Worsening in the absence of relapses was validated through primary record review.

**Results:**

Over 884 patient-years of follow-up, we identified 32 disability-worsening events in 31 patients. Notably, six events (18.7%) in five patients (1.9%) occurred in the absence of relapses. A positive association was identified between relapse trajectory and the hazard of disability worsening (hazard ratio (95% credible interval): 2.36 (1.61, 3.64)), and an inverse relationship with disability worsening occurring in the absence of relapses (0.41 (0.11, 1.47)).

**Conclusion:**

Irreversible disability worsening was rare in this proactively treated cohort of neuromyelitis optica spectrum disease patients (3.6 events per 100 patient-years). However, almost 19% of worsening occurred in the absence of relapses, reassuringly in only 2% of patients. Our findings support relapses as the principal driver of disability in neuromyelitis optica spectrum disease while also providing evidence of relapse-independent disability worsening beyond identifiable comorbidities.

## Introduction

Neuromyelitis optica spectrum disease (NMOSD) is a relapsing, autoimmune disease characterized by the presence of immunoglobulin G (IgG) antibodies targeting the astrocytic aquaporin-4 (AQP4) water channel in the central nervous system.^1^ NMOSD presents with severe attacks (relapses) which can result in irreversible astrocyte loss, demyelination and axonal damage.^2^ This damage leads to accumulation of disability over time. While relapse-related worsening is the primary mechanism of disability accumulation in NMOSD, emerging evidence reports that disability can also worsen gradually, in the absence of overt relapses.^3–8^ A recent study of 281 NMOSD patients from the National Cancer Center Registry in Korea identified seven patients (2.5%) who experienced disability worsening in the absence of relapse over a mean follow-up of 11.3 years.^9^ Although this proportion is small at the patient level, these events accounted for approximately 9% (16 of 171) of all disability-worsening events observed. In the phase III N-MOmentum trial, 56% (9 of 16) patients showed subclinical magnetic resonance imaging (MRI) activity within the spinal cord and elevated serum glial fibrillary acidic protein concentrations in the absence of clinical relapses.^6^ Among patients without relapses or changes in serum glial fibrillary acidic protein concentration, only 6% (9 of 143) showed spinal cord MRI activity.^6^ Accelerated brain or spinal cord atrophy has also been reported in clinically stable patients with NMOSD.^10,11^ Although less prevalent than in multiple sclerosis, confirmed disability worsening independent of relapses has been documented in 24.5% of patients with NMOSD at Massachusetts General Hospital between 2010 and 2020.^12^ The growing evidence of disability accumulation in the absence of relapses in NMOSD warrants an in-depth investigation into the intricate interplay between relapses and disability over time. Understanding the extent to which clinical relapses contribute to irreversible disability over the disease course is crucial, as it directly informs treatment strategies.

In this study, we aim to jointly evaluate individual relapse trajectories and irreversible disability in a large cohort of NMOSD patients, quantifying the role of relapses in disability worsening, with a particular emphasis on worsening occurring in the absence of relapses.

## Patients and methods

### Study population

Patients with AQP4-IgG-positive NMOSD, diagnosed by treating neurologists according to the 2015 International Consensus-Based Criteria,^1^ were identified from the international MSBase Registry (WHO International Clinical Trials Registry Platform ID ACTRN12605000455662). To minimize the risk of missing data, only patients who were followed up at least annually with a minimum of three Expanded Disability Status Scale (EDSS) scores were included. Data were extracted from MSBase in May 2023.

### Outcomes

A relapse was defined as the onset of new symptoms or worsening of existing symptoms lasting at least 24 h, provided it occurred at least 30 days after any previous relapse and was not attributable to concurrent illness or fever.

Disability accumulation was evaluated through sustained worsening in EDSS scores (increase in EDSS (ΔEDSS)) and confirmed disability worsening (CDW) events defined using a tiered approach. ΔEDSS was defined as any increase in EDSS score from the last assessment that persisted until the end of follow-up. Increases occurring within 12 months of a relapse were classified as relapse-associated, while those occurring more than 12 months after a relapse were considered relapse-independent. A sensitivity analysis using an 18-month window to define relapse-associated worsening was also performed.

CDW was defined as a sustained increase in EDSS score from a roving baseline (⩾1.5 step if baseline EDSS score was 0, or ⩾1.0 step if baseline EDSS score was 1.0–5.5, or ⩾0.5 step if baseline EDSS score was >5.5). The increase was required to be confirmed over at least 6 months and sustained throughout the remaining recorded follow-up. Disability worsening in the absence of relapses was defined using the same tiered EDSS increase relative to the roving baseline. In addition, the increase was required to occur in the absence of any relapses since the baseline EDSS assessment and to be confirmed without an intervening relapse. The baseline was reset after each relapse and confirmed worsening event. Disability-worsening events identified in the absence of relapses were validated using primary record verification.

Disability-worsening events were identified using R package MSoutcomes.

### Treatments

Immunotherapy was defined as treatment with azathioprine, cyclophosphamide, cyclosporin, mycophenolate mofetil, methotrexate, rituximab, eculizumab, tocilizumab, corticosteroids administered for at least 30 consecutive days, or combinations thereof. Use of multiple sclerosis disease-modifying therapies or intravenous immunoglobulin was not included. A relapse was considered untreated if it was not managed with corticosteroids or plasma exchange or intravenous immunoglobulin.

### Statistical analysis

We examined disability accumulation in relation to relapse and relapse-independent activity using two complementary outcomes: ΔEDSS and CDW events, both assessed as sustained worsening through the end of follow-up. ΔEDSS retains the magnitude of worsening and enables detection of subtle shifts in disability trajectories over time, whereas CDW prioritizes clinical robustness by capturing worsening of varying magnitudes relative to a roving baseline EDSS score. The use of both measures is particularly relevant in NMOSD, given the low numbers of CDW events reported in the literature.

We used a comprehensive joint modelling framework to evaluate the contribution of relapse trajectories to disability worsening. Relapses were modelled as a Poisson process with rate λ and time-to-disability worsening, either CDW or worsening in the absence of relapses, was modelled using a Cox proportional hazards model. The two models were linked with shared random effects.

In the *relapse trajectory submodel*, let denote the value of relapse count for patient i at time t . $x_{i}(t)$ is a vector of covariates including age at symptom onset, sex, race, topography of the presenting symptom, cumulative proportion of time on immunotherapies and time from first visit. $\lambda_{i}\left(t\right)$ represents the mean of the Poisson distribution, and $b_{i}$ is a patient-specific random effect accounting for within-subject correlation in the longitudinal counting process.

$$\log\left(\lambda_{i}\left(t\right)\right)=\beta x_{i}{}^{T}(t)+\;b_{i}$$

In the *time-to-disability worsening submodel*, let *h_i_ (t)* be the hazard function for patient i at time t and be the baseline hazard function. $z_{i}(t)$ is a vector of covariates including age at symptom onset, sex and topography of the presenting symptom. $b_{i}$ is the shared random effect joining the two submodels. The association parameter *α* quantifies the strength of the association between relapse trajectory and cumulative hazard of disability worsening.

$$h_{i}(t)=h_{0}\left(t\right)\;\exp\{\;\gamma z_{i}{}^{T}(t)+\alpha b_{i}\}$$

As a secondary analysis, we investigated the association between cumulative proportion of time on treatment with immunotherapy and the risk of CDW using a Cox proportional hazards model. The model was adjusted for age at symptom onset, sex, race, topography of the presenting symptom and proportion of untreated relapses. Potential multicollinearity among covariates was assessed prior to model fitting.

The models were fitted within a Bayesian framework using the integrated nested Laplace approximation implemented in R package INLA. For all regression coefficients, independent diffuse Gaussian priors (with mean 0, precision 1 × 10^−3^) were assumed.

## Results

A total of 261 patients with AQP4-IgG-positive NMOSD were included (Figure 1), of whom 219 were female. The median age at symptom onset was 32 years (quartiles: 23–47). At the first visit, median disease duration was 1.5 years (quartiles: 0.3–5.2) with a median EDSS score of 3.0 (quartiles: 2.0–4.5). Table 1 summarizes demographic and clinical characteristics of the study cohort. Patients excluded due to insufficient minimum follow-up were similar to those included in the study (eTable 1).

**Figure 1.**
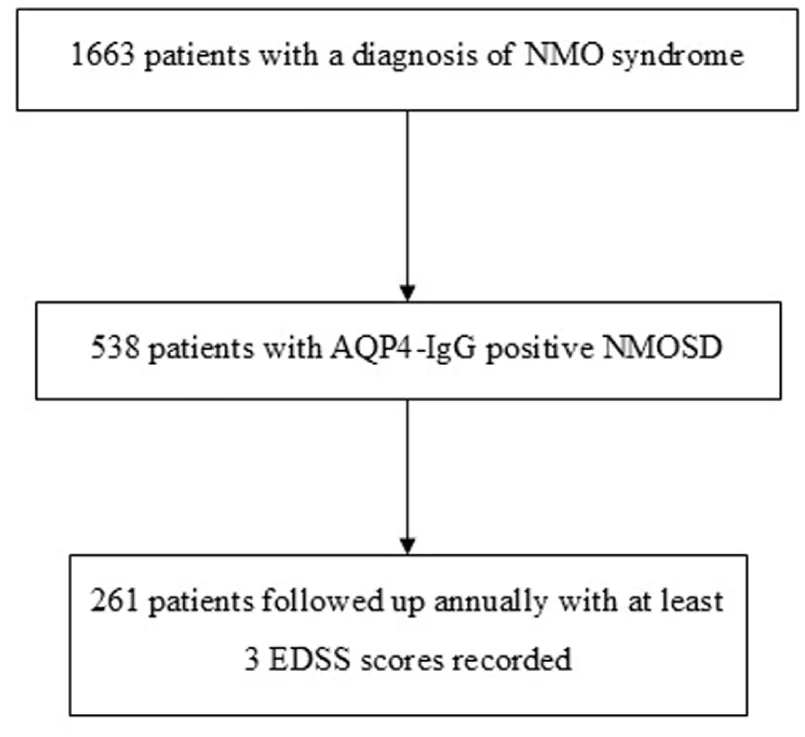
CONSORT chart of patient disposition.

**Table 1.** Description of the study cohort.

Characteristic	
Patients with AQP4-IgG-positive NMOSD, *n*	261
Antibody assays (centre level), *n* (%)	
Cell-based	15 (34.1)
ELISA	1 (2.3)
Indirect immunofluorescence	2 (4.5)
Cell-based or ELISA	1 (2.3)
Female sex, *n* (%)	219 (83.9)
Race, *n* (%)	
White	162 (62.1)
Middle Eastern	30 (11.5)
Asian	28 (10.7)
Black	5 (1.9)
Missing	36 (13.8)
Age at symptom onset,^a^ years	32 (23–47)
Topography of the presenting symptom,^b^ *n* (%)	
Spinal cord	109 (47.4)
Optic nerve	88 (38.3)
Brainstem	35 (15.2)
Supratentorial	30 (13.0)
Disease duration at the first visit,^a^ years	1.5 (0.3–5.2)
EDSS score at the first visit^a^	3.0 (2.0–4.5)
EDSS score at the last visit^a^	2.5 (1.5–5.0)
Annualized relapse rate during the follow-up^a^	0 (0.0–0.5)
Treatment exposure during the follow-up,^c^ *n* (%)	
Azathioprine	116 (44.4)
Rituximab	116 (44.4)
Prednisolone	57 (21.8)
Mycophenolate mofetil	17 (6.5)
Methotrexate	9 (3.4)
Cyclophosphamide	6 (2.3)
Eculizumab	1 (0.4)
Patients with no exposure to the treatments listed above during the follow-up	64 (24.5)
Recorded follow-up,^a^ years	2.48 (1.2–5.2)

a Median (first-third quartiles). ^b^Multiple topographies may be recorded per patient. ^c^Includes multiple treatment episodes of the same therapy per patient.

Among the included patients, 2509 visits with EDSS scores, spanning 884 patient-years, were analysed. A total of 279 relapses were identified in 120 patients during the credible period of visits with recorded EDSS assessments, with a median annualized relapse rate of 0 (quartiles: 0.0–0.5).

The stacked area chart (Figure 2) illustrates the mean cumulative increase in observed EDSS scores (ΔEDSS) over disease duration, distinguishing relapse-associated from relapse-independent contributions to overall all-cause disability worsening. At each time point, EDSS worsening was aggregated among patients experiencing sustained worsening and cumulatively summed over time. As the number of patients contributing data varied across time points, cumulative worsening is presented as the mean per patient, using the total number of patients who experienced sustained worsening as the denominator. The rug plot along the x-axis illustrates the density of relapses and EDSS assessments over time. As expected, most of the worsening occurred within 12 months of a relapse, with the contribution of relapses to overall disability being more pronounced during the early stages of the disease. However, the apparent increase over time in the mean cumulative worsening accrued in the absence of relapses within the preceding 12 months should be interpreted cautiously, given changes in the composition of patients contributing data across time points. In the sensitivity analysis, extending the post-relapse window to 18 months resulted in more relapse-associated worsening events. However, the relative contributions of relapse-associated and relapse-independent worsening to overall disability over time remained consistent with those observed using the 12-month window (eFigure 1).

**Figure 2.**
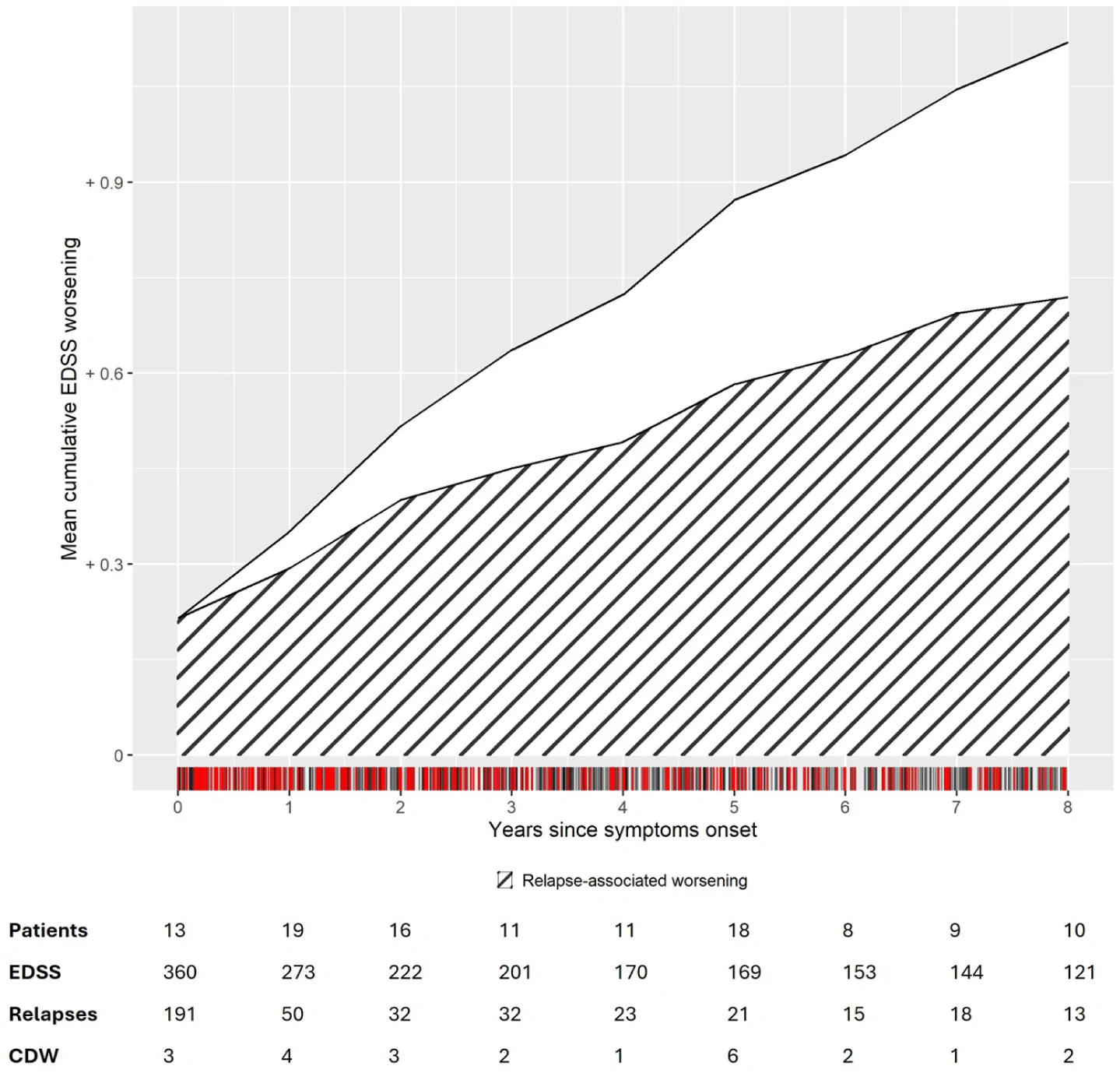
Cumulative worsening in EDSS scores (ΔEDSS) over disease duration. Stacked areas of relapse-associated and relapse-independent worsening are averaged by the total number of patients who experienced sustained worsening. Relapse-associated worsening was defined using a 12-month window. Rug marks along the x-axis indicate the timing of individual relapse events (red) and EDSS assessments (black). This figure represents an aggregated descriptive analysis and should not be interpreted as depicting individual patient trajectories or estimating the frequency of confirmed relapse-independent worsening or PIRA.

We identified 32 CDW events in 31 of the 261 patients (12%; 3.6 events per 100 patient-years). The association parameter of the joint model between relapse trajectory and CDW event provided strong evidence of association between relapses and CDW (hazard ratio (HR) (95% credible interval, CrI): 2.36 (1.61, 3.64)).

Of the 32 CDW events, 6 events (19%) occurring in 5 patients (0.68 events per 100 patient-years) were identified in the absence of relapses and verified through primary record review as true and not attributable to other medical conditions. Two additional events were excluded after medical review, as the worsening was not related to the absence of relapses but to other clinical conditions (eTable 2). The clinical profiles of the six events verified to have occurred in the absence of relapses are described in Table 2. The most commonly affected functional systems were cerebellar (67%), visual (50%), pyramidal (50%) and cerebral/cognitive (50%). Half of the events originated from a baseline EDSS score greater than 5.5, whereas the lowest baseline EDSS score associated with an event was 1.5. No disease activity on spinal cord and/or brain MRI was reported. Two events occurred during treatment with azathioprine, two during rituximab, and one while receiving mycophenolate mofetil. The association parameter of the joint model indicated a reduced hazard of these events with increasing relapse activity (HR (95% CrI): 0.41 (0.11, 1.47)), although the 95% CrI included the null value, likely reflecting limited statistical power due to the small number of events included in this analysis.

**Table 2.** Clinical profile of disability-worsening events occurred in the absence of relapses.

Patient	Worsening in EDSS scores (baseline to event)	Time (days) from baseline EDSS score to the worsening	Worsening in FSS (baseline to event)	Worsening in ambulation (baseline to event)	Age (years) at event	Disease duration (years) at event	Treatment at event	Duration (days) of sustained disability
1	4.0–6.0	540	Pyramidal: 1–2 Cerebellar: 0–3 Sensory: 1–2	Fully ambulatory to walking range < 100 m without assistance	70	5	Mycophenolate mofetil	1311
2	6.0–6.5	1862	Cerebellar: 1–3 Brainstem: 1–2 Visual: 2–3 Cerebral: 0–2	Walking range < 100 m without assistance to unilateral assistance, ⩾ 50 m	60	8	Untreated	266
3	7.0–7.5	396	Pyramidal: 4–5	Bilateral assistance, ⩾ 120 m to unilateral assistance, < 50 m	68	6	Azathioprine	995
3	7.5–8.0	657		Unilateral assistance, < 50 m to bilateral assistance, ⩾ 5 m, but < 120 m	70	8	Azathioprine	339
4	3.0–4.0	257	Cerebellar: 0–1 Visual: 4–6		34	4	Rituximab	588
5	1.5–3.0	270	Pyramidal: 0–2 Cerebellar: 0–1 Sensory: 1–2 Visual: 1–2		28	3	Rituximab	290

FSS: functional system score.

In the secondary analysis (Table 3), each 1% increase in the time on immunotherapies was associated with a 3% reduction in the hazard of CDW (HR (95% CrI): 0.97 (0.92, 0.99)).

**Table 3.** Multivariable analysis of the association between duration of immunotherapy and cumulative hazard of the confirmed disability worsening.

	Mean (95% credible interval)
Proportion of time treated with immunotherapies	−0.03 (−0.08, −0.006)
Sex	
Male (ref)	1.00
Female	0.26 (−2.65, 2.64)
Race	
White (ref)	1.00
Middle Eastern	4.20 (−0.26, 6.54)
Asian	4.94 (2.03, 6.89)
Black	−82.76 (−111.08, −53.60)
Missing	4.04 (1.79, 6.00)
Age at symptom onset, years	−0.09 (−0.19, 0.01)
Topography of the presenting symptom	
Spinal cord (ref)	1.00
Optic pathway	−1.28 (−3.99, 3.11)
Brainstem	−2.15 (−6.25, 4.61)
Mixed	−4.40 (−8.86, 3.73)
Unknown	−2.88 (−6.26, 4.16)
Proportion of relapses that were untreated, %	0.03 (0.01, 0.04)

## Discussion

In our cohort of 261 patients with NMOSD, irreversible accumulation of disability was predominantly relapse-driven. Only 2% of patients experienced worsening in the absence of relapses and without alternative explanations. However, at the event level, relapse-independent worsening accounted for a substantial proportion (19%) of all worsening events. The exploratory analysis of cumulative EDSS worsening suggested that an increasing proportion of disability accumulation over time was not attributable to overt relapses. Together, these findings provide a more nuanced, and hitherto underappreciated, view of disability accumulation in NMOSD, indicating that while relapses remain the principal driver, a non-negligible proportion of disability is contributed by relapse-independent worsening.

A longitudinal study including 466 adults with NMOSD from Taiwan reported a higher relapse rate in the first three years after diagnosis (0.67 (quartiles: 0.33, 1.20)), which declined markedly thereafter (0.00 (quartiles: 0.00, 0.12)).^13^ The German Neuromyelitis Optica Study Group (NEMOS) registry reported a similar trend, with the highest relapse rate in the first 2 years from disease onset, which then decreased by more than two-thirds, from 1.06 (95% confidence interval: 0.97, 1.16) to 0.33 (0.26, 0.43), over the subsequent two years and remained stable thereafter.^14^ A French multicentre study, similarly, reported a decline in the mean number of relapses after 2 years from onset.^15^ These observations align with our findings, which demonstrate that relapse-associated worsening is most pronounced in the early stages of the disease, while the relative contribution of relapse-independent disability accumulation appears to increase over time.

It is of interest that among the 279 relapses reported in our cohort, only 24 instances of related disability worsening were identified. This is at least partly attributable to our focus on disability worsening that remained sustained throughout the recorded follow-up. With proactive treatment, many NMOSD relapses are followed by at least partial resolution.^16^ The relapses reported here were associated with increases in disability that did not resolve during the available follow-up time.

In a cohort of 49 patients with AQP4-IgG-positive NMOSD from Massachusetts General Hospital, approximately one-fourth of the patients experienced relapse-independent CDW.^12^ This proportion was substantially lower than that reported in the matched cohort with multiple sclerosis, leading the authors to conclude that relapse-independent worsening is less common in NMOSD than in multiple sclerosis. In the N-MOmentum trial, 15% (27 of 174) of patients treated with inebilizumab experienced EDSS worsening, of whom 44.4% had no accompanying relapse. In the placebo arm, a higher proportion of patients (19 of 56) experienced EDSS worsening, of which 15.8% occurred in the absence of an accompanying relapse.^8^ It should be noted that adjudication of relapses in clinical trials may have influenced the reported incidence of relapse-independent worsening. In our cohort, only 2% of patients experienced CDW in the absence of relapses, consistent with recent data from observational studies, including findings from the MSBase registry.^5,9^ However, examination of these events provides a complementary perspective. Disability-worsening events not related to relapses accounted for almost 19% of all worsening events. Thus, relapse-independent mechanisms, while infrequent at the patient level, contribute meaningfully to the overall burden of disability.

A post hoc analysis of the phase 3 Prevention of Relapses in Neuromyelitis Optica (PREVENT) study estimated greater EDSS worsening in NMOSD patients who experienced two relapses compared to those with none.^17^ We confirm that disability worsening is associated with the trajectory of relapses over time, albeit not exclusively. Furthermore, a worsening of disability in the absence of relapses is less likely to occur among patients with frequent relapses. This is not surprising, as lower relapse activity may unmask underlying silent progression. Importantly, persistent treatment with immunotherapies is associated with a lower risk of CDW, which can presumably be attributed to their effect on reducing the risk of relapses.^18^

The clinical profile of the six worsening events that occurred in the absence of relapses or comorbid conditions in our cohort suggests that not all disability in NMOSD is accumulated through relapses. Although robust evidence supporting a truly progressive course in NMOSD is lacking, disability accrual in a small proportion of patients despite relapse suppression raises the possibility of non-relapsing mechanisms of central nervous system injury, such as chronic astrocytopathy or smouldering inflammatory processes. Non-NMOSD-specific contributors, including autoimmune comorbidities, may also contribute to or exacerbate functional deterioration. We have endeavoured to rule these contributions out by performing a thorough primary record verification on the cases with relapse-independent disability worsening. Age-related physiological decline and excess body weight further compound vulnerability to functional loss. In addition, delayed or slowly evolving sequelae of prior relapses may manifest as apparent new worsening, with incomplete recovery from earlier relapses resulting in incremental functional decline, or cumulative effects of prior inflammatory injury becoming clinically evident over time. This delayed consequence of prior relapses may potentially also be termed progression triggered by prior inflammatory events. Our approach to joint modelling is expected to account, at least in part, for these delayed effects of relapses on subsequent disability accumulation.

We cannot entirely rule out the possibility that milder, unrecorded relapses or subclinical changes on MRI may have contributed to disability worsening classified as relapse-independent. However, the requirement of at least one clinical visit per year lessens this risk. To further enhance specificity and minimize inter-rater variability in EDSS assessments, worsening events were required to be sustained until the end of follow-up. Likewise, the MSBase requirement for Neurostatus certification enhances the reliability and consistency of EDSS assessment across raters and sites. Finally, disability-worsening events were validated through primary record verification, in consultation with treating neurologists. The EDSS, although commonly used in NMOSD, does not adequately capture impairments related to the visual pathways or long tracts within the spinal cord, which are often prominent in these patients. Data from MRI and optical coherence tomography (OCT) were incomplete or unavailable and were not used to confirm relapses. However, if relapses were to be adjudicated by MRI, the proportion of disability not attributable to relapses may be higher. Data on body weight were also not consistently available, limiting our ability to evaluate whether body weight contributed to disability worsening in the absence of relapses through non-NMOSD-specific mechanisms. Although we included only AQP4-IgG-positive NMOSD patients, heterogeneity in diagnostic antibody assays across participating centres may have led to heterogeneity in diagnosis. A key strength of our study lies in the simultaneous modelling of the trajectory of relapses for each patient and their associated risk of disability worsening, addressing the highly heterogeneous presentation of NMOSD.

In conclusion, confirmed worsening of disability is rare in actively treated NMOSD. While relapses are the predominant driver of disability worsening in NMOSD, they are not the sole contributor. A small proportion of patients with NMOSD experience relapse-independent worsening, and the occurrence of such events appears to increase over time. Reassuringly, treatment with immunotherapy is associated with a reduced risk of disability progression of NMOSD, presumably mainly through reducing the risk of relapses. Further research is warranted to understand the pathophysiological mechanisms resulting in disability in NMOSD and its prevention.

## Supplemental Material

sj-docx-1-msj-10.1177_13524585261489039 – Supplemental material for How patients with neuromyelitis optica spectrum disease accumulate disability Supplemental material, sj-docx-1-msj-10.1177_13524585261489039 for How patients with neuromyelitis optica spectrum disease accumulate disability by Sifat Sharmin, Izanne Roos, Pakeeran Siriratnam, Serkan Ozakbas, Dana Horáková, Eva Kubala Havrdova, Petra Nytrová, Recai Turkoglu, Francesco Patti, Bassem Yamout, Samia J. Khoury, Abdullah Al-Asmi, Guy Laureys, Raed Alroughani, Jeannette Lechner-Scott, Cavit Boz, Ayse Altintas, Aysun Soysal, Sara Eichau, Anneke van der Walt, Vilija Jokubaitis, Helmut Butzkueven, Mastura Monif and Tomas Kalincik in Multiple Sclerosis Journal

## Data Availability

MSBase warehouse data from individual principal investigators who agree to share their datasets on a project-by-project basis. Each principal investigator will need to be approached individually for permission to access the datasets.
