# Supplementary material for: How patients with neuromyelitis optica spectrum disease accumulate disability
Source: Mult Scler. 2026 Oct 8;32(13):1421–30. doi: 10.1177/13524585261489039 (PMC13748495; doi:10.1177/13524585261489039)
Supplement: Supplementary file 1 [file sj-docx-1-msj-10.1177_13524585261489039.docx]

# **SUPPLEMENTARY MATERIAL**

**eTable 1 Description of the excluded cohort**

| **Characteristic** |  |
| --- | --- |
| Patients with AQP4-IgG positive NMOSD, n | 277 |
| Female sex, n (%) | 221 (79.8) |
| Age at symptom onset ^a^, year | 31 (24-42) |
| Disease duration at the first visit ^a^, year | 2.1 (0.5-5.7) |
| EDSS score at the first visit ^a^ | 2.0 (1.5-4.0) |
| EDSS score at the last visit ^a^ | 2.0 (1.5-4.0) |

^a^ median (first-third quartiles)

**eTable 2 Clinical profile of disability worsening events that were confirmed not due to absence of relapses, but other conditions**

| **Patient** | **Worsening in EDSS scores in absence of a recorded relapse (baseline to event)** | **Verified reason for worsening** |
| --- | --- | --- |
| 1 | 5.0 to 9.0 | Worsening occurred shortly after symptom onset, recorded during hospitalization for an unrelated reason. |
| 2 | 3.0 to 4.0 | Difficulty in walking unassisted for distances greater than 500 m, attributed to knee and back pain from osteoarthritis and lumbosacral radiculopathy. |

# **eTable 3 List of MSBase contributors**

The following contributors participated in data acquisition:

From S. Maria delle Croci Hospital, AUSL Romagna, Ravenna, Italy, Dr Matteo Foschi, Dr Andrea Surcinelli.

From New York University Langone Medical Center, New York, United States, Dr Ilya Kister.

From University of Debrecen, Debrecen, Hungary, Dr Tunde Csepany.

From PGIMER, Chandigarh, India, Dr Dheeraj Khurana.

From University of Queensland, Brisbane, Australia, Dr Pamela McCombe.

From Semmelweis University Budapest, Budapest, Hungary, Dr Magdolna Simo.

From Department of Neurology, Box Hill Hospital, Melbourne, Australia, Dr Katherine Buzzard, Dr Olga Skibina.

From Westmead Hospital, Sydney, Australia, Dr Justin Garber.

From Universidade Metropolitana de Santos, Santos, Brazil, Dr Yara Fragoso.

From Antwerp University Hospital, Edegem, Belgium, Dr Barbara Willekens.

From Neuro Rive-Sud, Quebec, Canada, Dr Francois Grand'Maison.

From Centre Hospitalier Universitaire de Sherbrooke, Canada, Dr Emmanuelle Lapointe.

From Università di Bologna, Bologna, Italy, Dr Alessandra Lugaresi, Dr Valentina Tomassini.

From King Fahad Specialist Hospital-Dammam, Khobar, Saudi Arabia, Dr Talal Al-Harbi.

From Flinders University, Adelaide, Australia, Dr Mark Slee.

From Concord Repatriation General Hospital, Sydney, Australia, Dr Todd A. Hardy, Dr Steve Vucic, Dr Stephen William Reddel, Dr Sudarshini Ramanathan.

From Kasr Al Ainy MS research Unit (KAMSU), Cairo, Egypt, Dr Nevin Shalaby.

From Hospital Universitario Virgen de Valme, Seville, Spain, Dr Ricardo Fernandez Bolaños.

From Hospital Universitario Donostia and IIS Biodonostia, San Sebastián, Spain, Dr Tamara Castillo-Triviño, Dr Javier Olascoaga.

From Hospital Germans Trias i Pujol, Badalona, Spain, Dr Cristina Ramo-Tello.

From Azienda Sanitaria Unica Regionale Marche - AV3, Macerata, Italy, Dr Elisabetta Cartechini, Dr Matteo Diamanti.

From Azienda Ospedaliera di Rilievo Nazionale San Giuseppe Moscati Avellino, Avellino, Italy, Dr Daniele Spitaleri.

From Kobe University Graduate School of Medicine, Division of Neurology, Kobe, Japan, Dr Riki Matsumoto.

From St. Michael's Hospital, Toronto, Canada, Dr Jiwon Oh.

From University Hospital Center Zagreb, Zagreb, Croatia, Dr Mario Habek.

From University of Szeged, Szeged, Hungary, Dr Cecilia Rajda.

From Bombay Hospital Institute of Medical Sciences, Mumbai, India, Dr Bhim Singhal.

From University of Florence, Florence, Italy, Dr Maria Pia Amato.

From Penang General Hospital, Penang, Malaysia, Dr Jyh Yung Hor.

From Royal Hospital, Muscat, Oman, Dr Jabir Alkhaboori.

From Hacettepe University, Ankara, Turkey, Dr Rana Karabudak.

From Brain and Mind Centre, Sydney, Australia, Dr Michael Barnett.

From Austin Health, Melbourne, Australia, Dr Richard Macdonell.

From CHUM MS Center and Universite de Montreal, Montreal, Canada, Dr Alexandre Prat, Dr Marc Girard, Dr Pierre Duquette, Dr Catherine Larochelle.

From CISSS Chaudière-Appalache, Levis, Canada, Dr Pierre Grammond.

From Hospital Clinic de Barcelona, Barcelona, Spain, Dr Yolanda Blanco.

From Petz A. County Hospital, Gyor, Hungary, Dr Gabor Rum.

From Razi University Hospital, Tunis, Tunisia, Dr Riadh Gouider, Dr Saloua Mrabet.

**MSBase Operations Team:** Ms Charlotte Sartori, Ms Pamela Farr, Ms Rein Moore, Mr Dusko Stupar, Ms Cynthia Tang, and Ms Sonya Smirnova.

**eFig 1 Cumulative worsening in EDSS scores (ΔEDSS) over disease duration**

Stacked areas of relapse-associated and relapse-independent worsening are averaged by the total number of patients experienced sustained worsening. Relapse-associated worsening was defined using an 18-month window.
